# Sexual Dimorphism in Hepatic, Adipose Tissue, and Peripheral Tissue Insulin Sensitivity in Obese Humans

**DOI:** 10.3389/fendo.2015.00182

**Published:** 2015-11-26

**Authors:** Kasper W. ter Horst, Pim W. Gilijamse, Barbara A. de Weijer, Murat Kilicarslan, Mariette T. Ackermans, Aart J. Nederveen, Max Nieuwdorp, Johannes A. Romijn, Mireille J. Serlie

**Affiliations:** ^1^Department of Endocrinology and Metabolism, Academic Medical Center, Amsterdam, Netherlands; ^2^Laboratory of Endocrinology, Department of Clinical Chemistry, Academic Medical Center, Amsterdam, Netherlands; ^3^Department of Radiology, Academic Medical Center, Amsterdam, Netherlands; ^4^Department of Vascular Medicine, Academic Medical Center, Amsterdam, Netherlands; ^5^Department of Medicine, Academic Medical Center, Amsterdam, Netherlands

**Keywords:** insulin resistance, liver fat, glucose disposal, obesity, sexual dimorphism, diabetes

## Abstract

Glucose and lipid metabolism differ between men and women, and women tend to have better whole-body or muscle insulin sensitivity. This may be explained, in part, by differences in sex hormones and adipose tissue distribution. Few studies have investigated gender differences in hepatic, adipose tissue, and whole-body insulin sensitivity between severely obese men and women. In this study, we aimed to determine the differences in glucose metabolism between severely obese men and women using tissue-specific measurements of insulin sensitivity. Insulin sensitivity was compared between age and body mass index (BMI)-matched obese men and women by a two-step euglycemic hyperinsulinemic clamp with infusion of [6,6-^2^H_2_]glucose. Basal endogenous glucose production (EGP) and insulin sensitivity of the liver, adipose tissue, and peripheral tissues were assessed. Liver fat content was assessed by proton magnetic resonance spectroscopy in a subset of included subjects. We included 46 obese men and women (age, 48 ± 2 vs. 46 ± 2 years, *p* = 0.591; BMI, 41 ± 1 vs. 41 ± 1 kg/m^2^, *p* = 0.832). There was no difference in basal EGP (14.4 ± 1.0 vs. 15.3 ± 0.5 μmol · kg fat-free mass^−1^ · min^−1^, *p* = 0.410), adipose tissue insulin sensitivity (insulin-mediated suppression of free fatty acids, 71.6 ± 3.6 vs. 76.1 ± 2.6%, *p* = 0.314), or peripheral insulin sensitivity (insulin-stimulated rate of disappearance of glucose, 26.2 ± 2.1 vs. 22.7 ± 1.7 μmol · kg^−1^ · min^−1^, *p* = 0.211). Obese men were characterized by lower hepatic insulin sensitivity (insulin-mediated suppression of EGP, 61.7 ± 4.1 vs. 72.8 ± 2.5% in men vs. women, respectively, *p* = 0.028). Finally, these observations could not be explained by differences in liver fat content (men vs. women, 16.5 ± 3.1 vs. 16.0 ± 2.5%, *p* = 0.913, *n* = 27). We conclude that obese men have lower hepatic, but comparable adipose tissue and peripheral tissue, insulin sensitivity compared to similarly obese women. Hepatic insulin resistance may contribute to the higher prevalence of diabetes in obese men. Further insight into the mechanisms underlying this gender difference may reveal novel targets for diabetes prevention and/or therapy.

## Introduction

Obesity is the most important risk factor for insulin resistance and type 2 diabetes (1), and an increasing threat to both men and women worldwide (2). It is, however, becoming increasingly evident that handling of nutrients (3, 4), metabolic adaptations to overnutrition (5, 6), and the global prevalence of clinically overt dyslipidemia and diabetes (7, 8) are different for the two sexes. These observations, in addition to concerns about the underrepresentation of women in clinical research (3, 9), have fueled recent interest into sexual dimorphism in metabolism and metabolic disorders.

Insulin resistance is the major contributor to cardiometabolic complications of obesity, including diabetes (10, 11). Several studies have investigated whole-body and/or muscle insulin sensitivity in men and women [recently reviewed by Lundsgaard and Kiens (12)]. Most (13–19), but not all (20, 21), of the available evidence from euglycemic hyperinsulinemic clamp or arterial–venous balance studies suggests that insulin-stimulated glucose uptake is higher in women than men, indicating that women generally show higher peripheral insulin sensitivity. This may be related to a more favorable adipose tissue distribution in women [more subcutaneous and less visceral fat compared to men (4)] as well as to levels of circulating sex hormones, with estrogen having insulin-sensitizing (12) and anti-inflammatory (22) properties. This is strengthened by the findings that postmenopausal women rapidly gain visceral fat and become less insulin sensitive (23), while estrogen administration to postmenopausal women improves insulin action (24). By contrast, administration of gonadotropin-releasing hormone agonists (to induce short-term hypogonadism) or testosterone does not affect whole-body insulin sensitivity in healthy men (25).

Few studies have investigated insulin sensitivity in matched severely obese men and women, who are exceptionally prone to develop insulin resistance (26), or described gender differences in tissue-specific measurements of insulin sensitivity in liver and adipose tissue of men and women. Novel insight into to pathophysiological mechanisms underlying these differences in metabolism may reveal novel targets as well as promote more personalized therapeutic strategies.

In the present study, we aimed to determine the gender differences in glucose metabolism and tissue-specific insulin action using detailed metabolic tracer studies in a cohort of severely obese men and women.

## Materials and Methods

### Subjects

To assess the sex-specific differences in glucose metabolism and insulin action, we selected age and body mass index (BMI)-matched men and women (*n* = 46) from a previously described cohort of obese men and women (27). Subjects were eligible for the present analysis if they were severely obese (BMI >35 kg/m^2^) and had stable weight (<5% weight change) for 3 months prior to the study. Subjects were excluded in case of a history of insulin-dependent diabetes, use of alcohol (>2 U/day) or recreational drugs, use of psychoactive medication, or any somatic disorder except for obesity-related conditions (e.g., dyslipidemia, hypertension, or impaired glucose tolerance).

Subjects completed a medical evaluation including medical history, physical examination, blood tests, and assessment of body composition by bioelectrical impedance analysis (Maltron BF-906, Rayleigh, UK). On a separate visit, subjects underwent a two-step euglycemic hyperinsulinemic clamp for the assessment of glucose metabolism and insulin sensitivity.

All procedures were approved by the Academic Medical Center medical ethics committee and all subjects provided written informed consent in accordance with the Declaration of Helsinki.

### Experimental Protocol

Glucose clamp studies were performed according to standard operating procedures, which have been described in detail elsewhere (27). Briefly, the basal rate of endogenous glucose production (EGP), hepatic insulin sensitivity (expressed as the insulin-mediated suppression of basal EGP), adipose tissue insulin sensitivity [expressed as the insulin-mediated suppression of circulating free fatty acids (FFA)], and peripheral insulin sensitivity [expressed as the insulin-stimulated rate of disappearance (Rd) of glucose] were assessed after an overnight fast during a two-step euglycemic hyperinsulinemic clamp with infusion of [6,6-^2^H_2_]glucose as glucose tracer.

After 2 h of tracer equilibration, insulin infusion was started for 2 h per step at a rate of 20 and 60 mU · m^−2^ · min^−1^ during step 1 and 2, respectively. Plasma glucose was maintained at 5.0 mmol/l by infusion of exogenous glucose enriched with [6,6-^2^H_2_]glucose. Three (after tracer equilibration) or five (after each 2-h step of insulin infusion) blood samples with a 5-min interval were drawn to assess tracer enrichment for calculation of EGP and Rd and for measurements of glucoregulatory hormones.

Plasma glucose, glucoregulatory hormones, enrichment of [6,6^2^H_2_]glucose (tracer-to-tracee ratio), and lipids were determined, as previously described (27). EGP and Rd were calculated using modified versions of the Steele equations for the steady state (basal EGP) or non-steady state (during insulin infusion), and expressed as μmol · [kg fat-free mass (FFM)]^−1^ · min^−1^ and μmol · (kg body weight)^−1^ · min^−1^, respectively (28, 29).

In a subset of men (*n* = 8) and women (*n* = 19), intrahepatic triglyceride (IHTG) content was assessed by proton magnetic resonance spectroscopy (^1^H-MRS) on the morning of the clamp after an overnight fast. Liver ^1^H-MRS spectra were obtained, as previously described (30), and IHTG content was defined as the percentage of liver volume comprised of fat.

### Statistical Analysis

Data are expressed as mean ± standard error of the mean (SEM), unless stated otherwise. Groups were compared by two-tailed independent samples *t*-test. Correlations were evaluated by Pearson’s correlation coefficient. Findings were considered significant if the *p*-value was <0.05. Analyses were performed using IBM SPSS Statistics 22 (Armonk, NY, USA).

## Results

We included 46 age and BMI-matched severely obese men and women (Table 1). Men and women were well-matched in terms of age, BMI, and most other baseline characteristics, but women had higher total body fat content and high-density lipoprotein cholesterol (HDL). Fasting plasma glucose and insulin levels did not differ between men and women. Two obese women were postmenopausal. One woman had (non-insulin-dependent) type 2 diabetes and was treated with oral hypoglycemic agents.

**Table 1:** **Baseline characteristics of included men and women**.

	Men	Women	*p*
*N*	23	23	–
Age (years)	48 ± 2	46 ± 2	0.591
BMI (kg/m^2^)	41 ± 1	41 ± 1	0.832
Body fat content (%)	43 ± 2	52 ± 1	<0.001
Fasting glucose (mmol/l)	5.5 ± 0.1	5.6 ± 0.2	0.728
Fasting insulin (pmol/l)	105 ± 9	103 ± 9	0.882
Triglycerides (mmol/l)	1.5 ± 0.2	1.3 ± 0.1	0.228
Cholesterol (mmol/l)	4.5 ± 0.3	4.9 ± 0.2	0.289
LDL (mmol/l)	3.1 ± 0.1	3.2 ± 0.2	0.795
HDL (mmol/l)	1.0 ± 0.1	1.1 ± 0.1	0.039

*Data are mean ± SEM*.

**Figure 1:**
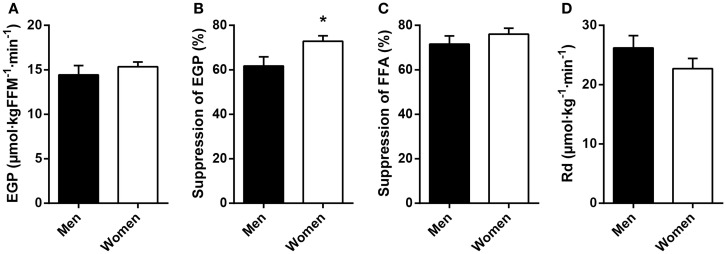
**Basal glucose metabolism and tissue-specific measurements of insulin action in age and BMI-matched severely obese men and women**. **(A)** Endogenous glucose production (EGP) was assessed in the basal (fasted) state. **(B)** Hepatic insulin sensitivity was defined as the relative suppression of basal EGP by insulin during step 1 of the clamp. **(C)** Adipose tissue insulin sensitivity was defined as the relative suppression of circulating free fatty acids (FFA) by insulin during step 1 of the clamp. **(D)** Peripheral insulin sensitivity was defined as the insulin-stimulated rate of disappearance (Rd) of glucose during step 2 of the clamp. Data are mean ± SEM. *p = 0.028.

Fasting plasma FFA levels were higher in women (Table 2). The basal rate of EGP did not differ between men and women (Figure 1A), but men had markedly lower insulin-mediated suppression of EGP (Figure 1B), indicating that severely obese men are characterized by lower hepatic insulin sensitivity compared to similarly obese women. Plasma glucagon levels during step 1 of the clamp were also higher in men (Table 2). Insulin-mediated suppression of FFA (Figure 1C) and insulin-stimulated Rd of glucose (Figure 1D) were not different between both groups, indicating that severely obese men and women are characterized by similar adipose tissue and peripheral tissue insulin sensitivity. We recently defined cutoff values for euglycemic hyperinsulinemic clamp-derived insulin resistance (27), and 91% of included subjects had Rd < 37.3 μmol · kg^−1^ · min^−1^, indicative of insulin resistance. Detailed data from the two-step euglycemic hyperinsulinemic clamp studies are presented in Table 2.

**Table 2:** **Metabolic parameters and fluxes during two-step euglycemic hyperinsulinemic clamp studies (*n* = 46)**.

	Men	Women	*p*
**Basal**
Glucose (mmol/l)	5.5 ± 0.1	5.6 ± 0.2	0.728
Insulin (pmol/l)	105 ± 9	103 ± 9	0.882
Glucagon (ng/l)	83 ± 5	66 ± 4	0.011
Cortisol (nmol/l)	300 ± 36	241 ± 22	0.153
EGP (μmol · kgFFM^−1^ · min^−1^)	14.4 ± 1.0	15.3 ± 0.5	0.410
FFA (mmol/l)	0.57 ± 0.03	0.77 ± 0.03	<0.001
**Clamp, step 1**
Insulin (pmol/l)	255 ± 18	278 ± 18	0.359
Glucagon (ng/l)	76 ± 5	60 ± 4	0.018
Cortisol (nmol/l)	235 ± 19	295 ± 25	0.095
Suppression of EGP (%)	61.7 ± 4.1	72.8 ± 2.5	0.028
Suppression of FFA (%)	71.6 ± 3.6	76.1 ± 2.6	0.314
**Clamp, step 2**
Insulin (pmol/l)	723 ± 38	740 ± 38	0.749
Glucagon (ng/l)	61 ± 4	52 ± 5	0.163
Cortisol (nmol/l)	291 ± 34	278 ± 28	0.770
Rd (μmol · kg^−1^ · min^−1^)	26.2 ± 2.1	22.7 ± 1.7	0.211
Rd (μmol · kgFFM^−1^ · min^−1^)	48.8 ± 7.3	47.2 ± 3.3	0.846

*Data are mean ± SEM. Basal, after an overnight fast; step 1, after 2 h of low-dose insulin infusion; step 2, after 2 h of high-dose insulin infusion*.

**Figure 2:**
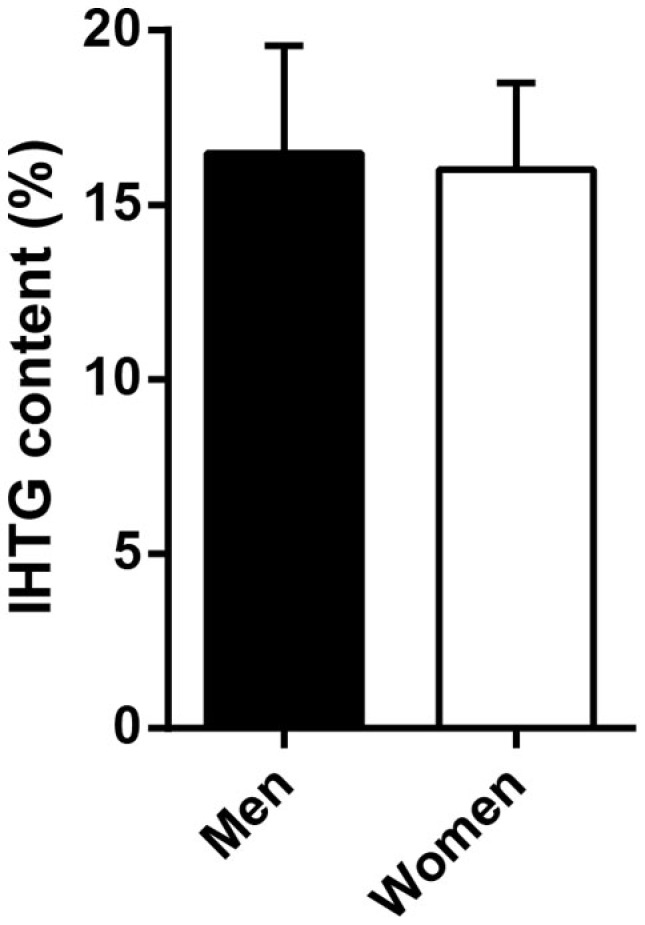
**Intrahepatic triglyceride (IHTG) content in a subset of severely obese men and women**. Men (*n* = 8) and women (*n* = 19) did not differ in IHTG content by ^1^H-MRS. Data are mean ± SEM.

Differences in insulin sensitivity could not be explained by differences in (low-grade) systemic inflammation (C-reactive protein, men vs. women, 7.1 ± 2.6 vs. 11.6 ± 2.6 mg/l, *p* = 0.244) or liver fat content (Figure 2). Plasma glucagon levels were not correlated to EGP or hepatic insulin sensitivity during the basal state or step 1 of the clamp in men, women, or all subjects (not shown).

## Discussion

We show that sexual dimorphism in metabolism is present in severely obese men and women. The current experimental protocol allowed us to quantitatively determine the degree of insulin resistance in the liver, adipose tissue, and muscle. Our results show that, in an age and BMI-matched cohort of severely obese men and women, men have comparable peripheral insulin sensitivity, but lower hepatic insulin sensitivity compared to women. In addition, women have higher fasting plasma levels of FFA, whereas insulin-mediated suppression of FFA levels is similar to men.

Although several previous studies suggest that women may physiologically be inclined to have better muscle insulin sensitivity (13–17), both men and women in the present study were severely obese and characterized by moderate-to-severe peripheral insulin resistance compared to a non-obese reference population (27). Our data, in line with one previous study of obese individuals (21), suggest that the insulin-sensitizing effects of female sex may be blunted in the context of severe obesity, or that insulin-desensitizing mechanisms in severe obesity may overwhelm the protective mechanisms that exist in lean women.

In the basal state and during prolonged fasting, women rely on lipid oxidation for energy more than men (31). Women generally have higher basal rates of lipolysis, resulting in higher circulating levels of FFA (20). In accordance, we also show that severely obese women have higher fasting plasma FFA levels. Notably, infusion of insulin, the major inhibitory regulator of lipolysis, and circulating FFA levels (31), resulted in similar suppression of FFA in men and women, suggesting that insulin-mediated inhibition of adipose tissue lipolysis does not exhibit sexual dimorphism in obese subjects.

Several mechanisms may explain the difference in hepatic insulin sensitivity between obese men and women. Although the present study did not address the molecular pathophysiology, we did not observe any gender differences in circulating levels of C-reactive protein or IHTG content. These findings may suggest that differences in systemic inflammation or hepatic steatosis, both often implicated in the development of hepatic insulin resistance (32), did not contribute to the observed sexual dimorphism. Nevertheless, we did not measure local inflammatory signals in the liver nor individual hepatic lipid species, such as diacylglycerol (33), which may have contributed to hepatic insulin resistance in obese men. In fact, estrogen reduces hepatic diacylglycerol accumulation and improves hepatic insulin resistance in mice (34, 35). Estrogen and estrogen receptor β agonists might thus be considered novel therapeutic targets for hepatic insulin resistance and/or steatosis (36, 37).

Since glucagon stimulates EGP (38), higher levels of glucagon during the first step of the clamp may have contributed to the lower insulin-mediated suppression of EGP in obese men. However, since the absolute gender difference in glucagon was small and glucagon levels were not directly correlated to EGP or insulin-mediated suppression of EGP in the present study, further research is required to fully elucidate the role of glucagon in explaining sexual dimorphism in metabolism. Other mechanisms, such as sex-specific differences in visceral adipose tissue-derived lipolysis and fatty acid flux (39, 40) or differences in unmeasured glucoregulatory hormones such as growth hormone (41), may also have contributed to the observed difference in hepatic insulin resistance. Lastly, we did not measure specific adipokines, but it has been shown that circulating adiponectin is lower in men (42). Since adiponectin reduces EGP in mice (43), lower levels may be implicated in elevated EGP and/or hepatic insulin resistance.

The observation that severely obese men are prone to develop hepatic insulin resistance is also of clinical relevance. Failure of insulin to appropriately suppress EGP, i.e., hepatic insulin resistance, is an important contributor to the development of hyperglycemia and (pre)diabetes (44–46). A male predisposition to develop obesity-related hepatic insulin resistance may thus contribute to the higher global prevalence of diabetes in men (7). In accordance, prevention of type 2 diabetes by metformin, an oral hypoglycemic agent that primarily targets hepatic insulin resistance (47), appeared to be most effective in obese and male prediabetes patients (48). Further understanding of the precise metabolic defects in (obese) men and women will help to personalize treatment strategies.

We acknowledge that the contribution of adipose tissue glucose uptake to whole-body insulin-stimulated glucose uptake has not been clearly established, and estimates range from ~4 to 33% of total glucose flux (49, 50). Differences in body composition [i.e., women typically have twice the adipose tissue mass compared to men with the same BMI (4, 12)] may thus influence study results when metabolic fluxes, such as the Rd of glucose, are expressed per kilogram of body weight or kilogram of fat-free mass. In the present study, however, Rd was comparable for severely obese men and women when expressed per kilogram of body weight (Figure 1D) or fat-free body mass (Table 2), suggesting that differences in body fat content between men and women did not influence the main findings.

Unfortunately, we did not have data on the menstrual status of included women and, therefore, could not take this into account. Women generally do not undergo major changes in peripheral insulin sensitivity throughout the menstrual cycle (12), but plasma sex hormones may vary considerably, and we cannot rule out an effect of menstrual status on measurements of hepatic and/or adipose tissue insulin resistance. We also acknowledge that glucose tolerance and HbA_1c_ tests were not performed in the present study. We thus cannot rule out that some individuals with normal fasting glucose would otherwise be diagnosed with impaired glucose tolerance or type 2 diabetes on the basis of those tests. Finally, sensitivity analysis did not reveal any differences in our results when all analyses were repeated excluding two postmenopausal women and/or one woman with pre-existing type 2 diabetes.

In conclusion, we show that insulin sensitivity of adipose tissue and peripheral tissues is similarly impaired in severely obese men and women, but these men are characterized by lower hepatic insulin sensitivity. A predisposition to develop obesity-related hepatic insulin resistance may contribute to the higher prevalence of diabetes in obese men. Further studies are required to elucidate the molecular mechanisms underlying the observed sexual dimorphism in obesity-related insulin resistance.

## Author Contributions

KH acquired data, performed the analyses, and drafted the manuscript. PG and BW acquired data and critically revised the manuscript. MA was responsible for laboratory analyses and critically revised the manuscript. AN was responsible for ^1^H-MRS experiments and critically revised the manuscript. MK, MN, JR, and MS contributed to interpretation of the results and critically revised the manuscript. All authors reviewed and approved the final manuscript.

## Conflict of Interest Statement

The authors declare that the research was conducted in the absence of any commercial or financial relationships that could be construed as a potential conflict of interest.

## Abbreviations

^1^H-MRS, proton magnetic resonance spectroscopy; BMI, body mass index; EGP, endogenous glucose production; FFA, free fatty acids; FFM, fat-free mass; HDL, high-density lipoprotein cholesterol; LDL, low-density lipoprotein cholesterol; Rd, rate of disappearance (of glucose); SEM, standard error of the mean.

## References

[1] Sullivan PW Morrato EH Ghushchyan V Wyatt HR Hill JO. Obesity, inactivity, and the prevalence of diabetes and diabetes-related cardiovascular comorbidities in the U.S., 2000-2002. Diabetes Care (2005) 28:1599–603. 10.2337/diacare.28.7.1599 15983307

[2] Zimmet P Alberti KG Shaw J. Global and societal implications of the diabetes epidemic. Nature (2001) 414:782–7. 10.1038/414782a 11742409

[3] Varlamov O Bethea CL Roberts CT Jr Sex-specific differences in lipid and glucose metabolism. Front Endocrinol (2014) 5:241 10.3389/fendo.2014.00241 PMC4298229 25646091

[4] Santosa S Jensen M. The sexual dimorphism of lipid kinetics in humans. Front Endocrinol (2015) 6:103. 10.3389/fendo.2015.00103 26191040 PMC4489151

[5] Peterson LR Soto PF Herrero P Mohammed BS Avidan MS Schechtman KB Impact of gender on the myocardial metabolic response to obesity. JACC Cardiovasc Imaging (2008) 1:424–33. 10.1016/j.jcmg.2008.05.004 19356462 PMC2982260

[6] Palmer BF Clegg DJ. The sexual dimorphism of obesity. Mol Cell Endocrinol (2015) 402:113–9. 10.1016/j.mce.2014.11.029 25578600 PMC4326001

[7] Wild S Roglic G Green A Sicree R King H. Global prevalence of diabetes: estimates for the year 2000 and projections for 2030. Diabetes Care (2004) 27:1047–53. 10.2337/diacare.27.5.1047 15111519

[8] Goff DC Jr Bertoni AG Kramer H Bonds D Blumenthal RS Tsai MY Dyslipidemia prevalence, treatment, and control in the multi-ethnic study of atherosclerosis (MESA): gender, ethnicity, and coronary artery calcium. Circulation (2006) 113:647–56. 10.1161/circulationaha.105.552737 16461837

[9] Institute of Medicine (US) Board on Population Health and Public Health Practice. Sex-Specific Reporting of Scientific Research: A Workshop Summary. Washington, DC: National Academies Press (US) (2012). 72 p. 22379657

[10] Kahn SE Hull RL Utzschneider KM. Mechanisms linking obesity to insulin resistance and type 2 diabetes. Nature (2006) 444:840–6. 10.1038/nature05482 17167471

[11] Accili D. Lilly lecture 2003: the struggle for mastery in insulin action: from triumvirate to republic. Diabetes (2004) 53:1633–42. 10.2337/diabetes.53.7.1633 15220184

[12] Lundsgaard AM Kiens B. Gender differences in skeletal muscle substrate metabolism – molecular mechanisms and insulin sensitivity. Front Endocrinol (2014) 5:195. 10.3389/fendo.2014.00195 25431568 PMC4230199

[13] Karakelides H Irving BA Short KR O’Brien P Nair KS. Age, obesity, and sex effects on insulin sensitivity and skeletal muscle mitochondrial function. Diabetes (2010) 59:89–97. 10.2337/db09-0591 19833885 PMC2797949

[14] Hoeg L Roepstorff C Thiele M Richter EA Wojtaszewski JF Kiens B. Higher intramuscular triacylglycerol in women does not impair insulin sensitivity and proximal insulin signaling. J Appl Physiol (2009) 107:824–31. 10.1152/japplphysiol.91382.2008 19574502

[15] Vistisen B Hellgren LI Vadset T Scheede-Bergdahl C Helge JW Dela F Effect of gender on lipid-induced insulin resistance in obese subjects. Eur J Endocrinol (2008) 158:61–8. 10.1530/eje-07-0493 18166818

[16] Borissova AM Tankova T Kirilov G Koev D. Gender-dependent effect of ageing on peripheral insulin action. Int J Clin Pract (2005) 59:422–6. 10.1111/j.1368-5031.2005.00209.x 15853858

[17] Sumner AE Kushner H Sherif KD Tulenko TN Falkner B Marsh JB. Sex differences in African-Americans regarding sensitivity to insulin’s glucoregulatory and antilipolytic actions. Diabetes Care (1999) 22:71–7. 10.2337/diacare.22.1.71 10333906

[18] Hoeg LD Sjoberg KA Jeppesen J Jensen TE Frosig C Birk JB Lipid-induced insulin resistance affects women less than men and is not accompanied by inflammation or impaired proximal insulin signaling. Diabetes (2011) 60:64–73. 10.2337/db10-0698 20956497 PMC3012198

[19] Paula FJ Pimenta WP Saad MJ Paccola GM Piccinato CE Foss MC. Sex-related differences in peripheral glucose metabolism in normal subjects. Diabete Metab (1990) 16:234–9. 2210019

[20] Soeters MR Sauerwein HP Groener JE Aerts JM Ackermans MT Glatz JF Gender-related differences in the metabolic response to fasting. J Clin Endocrinol Metab (2007) 92:3646–52. 10.1210/jc.2007-0552 17566089

[21] Koska J Stefan N Permana PA Weyer C Sonoda M Bogardus C Increased fat accumulation in liver may link insulin resistance with subcutaneous abdominal adipocyte enlargement, visceral adiposity, and hypoadiponectinemia in obese individuals. Am J Clin Nutr (2008) 87:295–302. 18258617 10.1093/ajcn/87.2.295

[22] Shen M Kumar SP Shi H. Estradiol regulates insulin signaling and inflammation in adipose tissue. Horm Mol Biol Clin Investig (2014) 17:99–107. 10.1515/hmbci-2014-0007 25372734 PMC4221806

[23] Polotsky HN Polotsky AJ Metabolic implications of menopause. Semin Reprod Med (2010) 28:426–34. 10.1055/s-0030-1262902 20865657

[24] Van Pelt RE Gozansky WS Schwartz RS Kohrt WM. Intravenous estrogens increase insulin clearance and action in postmenopausal women. Am J Physiol Endocrinol Metab (2003) 285:E311–7. 10.1152/ajpendo.00490.2002 12684221 PMC2819703

[25] Host C Gormsen LC Hougaard DM Christiansen JS Pedersen SB Gravholt CH. Acute and short-term chronic testosterone fluctuation effects on glucose homeostasis, insulin sensitivity, and adiponectin: a randomized, double-blind, placebo-controlled, crossover study. J Clin Endocrinol Metab (2014) 99:E1088–96. 10.1210/jc.2013-2807 24606070

[26] Ferrannini E Natali A Bell P Cavallo-Perin P Lalic N Mingrone G Insulin resistance and hypersecretion in obesity. European Group for the Study of Insulin Resistance (EGIR). J Clin Invest (1997) 100:1166–73. 10.1172/jci119628 9303923 PMC508292

[27] Ter Horst KW Gilijamse PW Koopman KE de Weijer BA Brands M Kootte RS Insulin resistance in obesity can be reliably identified from fasting plasma insulin. Int J Obes (2015). 10.1038/ijo.2015.125 26155920

[28] Finegood DT Bergman RN Vranic M Estimation of endogenous glucose production during hyperinsulinemic-euglycemic glucose clamps. Comparison of unlabeled and labeled exogenous glucose infusates. Diabetes (1987) 36:914–24. 10.2337/diab.36.8.914 3297886

[29] Steele R Influences of glucose loading and of injected insulin on hepatic glucose output. Ann N Y Acad Sci (1959) 82:420–30. 10.1111/j.1749-6632.1959.tb44923.x 13833973

[30] van der Valk F Hassing C Visser M Thakkar P Mohanan A Pathak K The effect of a diiodothyronine mimetic on insulin sensitivity in male cardiometabolic patients: a double-blind randomized controlled trial. PLoS One (2014) 9:e86890. 10.1371/journal.pone.0086890 24586256 PMC3931609

[31] Hedrington MS Davis SN. Sexual dimorphism in glucose and lipid metabolism during fasting, hypoglycemia, and exercise. Front Endocrinol (2015) 6:61. 10.3389/fendo.2015.00061 25964778 PMC4410598

[32] Perry RJ Samuel VT Petersen KF Shulman GI. The role of hepatic lipids in hepatic insulin resistance and type 2 diabetes. Nature (2014) 510:84–91. 10.1038/nature13478 24899308 PMC4489847

[33] Samuel VT Liu ZX Wang A Beddow SA Geisler JG Kahn M Inhibition of protein kinase C epsilon prevents hepatic insulin resistance in nonalcoholic fatty liver disease. J Clin Invest (2007) 117:739–45. 10.1172/jci30400 17318260 PMC1797607

[34] Zhu L Brown WC Cai Q Krust A Chambon P McGuinness OP Estrogen treatment after ovariectomy protects against fatty liver and may improve pathway-selective insulin resistance. Diabetes (2013) 62:424–34. 10.2337/db11-1718 22966069 PMC3554377

[35] Zhu L Martinez MN Emfinger CH Palmisano BT Stafford JM. Estrogen signaling prevents diet-induced hepatic insulin resistance in male mice with obesity. Am J Physiol Endocrinol Metab (2014) 306:E1188–97. 10.1152/ajpendo.00579.2013 24691030 PMC4116406

[36] Jelenik T Roden M How estrogens prevent from lipid-induced insulin resistance. Endocrinology (2013) 154:989–92. 10.1210/en.2013-1112 23429711

[37] Alonso-Magdalena P Ropero AB Garcia-Arevalo M Soriano S Quesada I Muhammed SJ Antidiabetic actions of an estrogen receptor beta selective agonist. Diabetes (2013) 62:2015–25. 10.2337/db12-1562 23349481 PMC3661616

[38] Matsuda M Defronzo RA Glass L Consoli A Giordano M Bressler P Glucagon dose-response curve for hepatic glucose production and glucose disposal in type 2 diabetic patients and normal individuals. Metabolism (2002) 51:1111–9. 10.1053/meta.2002.34700 12200754

[39] Mittelman SD Bergman RN. Inhibition of lipolysis causes suppression of endogenous glucose production independent of changes in insulin. Am J Physiol Endocrinol Metab (2000) 279:E630–7. 10950832 10.1152/ajpendo.2000.279.3.E630

[40] Mittendorfer B. Origins of metabolic complications in obesity: adipose tissue and free fatty acid trafficking. Curr Opin Clin Nutr Metab Care (2011) 14:535–41. 10.1097/MCO.0b013e32834ad8b6 21849896 PMC3711689

[41] Jeffcoate W. Growth hormone therapy and its relationship to insulin resistance, glucose intolerance and diabetes mellitus: a review of recent evidence. Drug Saf (2002) 25:199–212. 10.2165/00002018-200225030-00005 11945115

[42] Hoeg LD Sjoberg KA Lundsgaard AM Jordy AB Hiscock N Wojtaszewski JF Adiponectin concentration is associated with muscle insulin sensitivity, AMPK phosphorylation, and ceramide content in skeletal muscles of men but not women. J Appl Physiol (2013) 114:592–601. 10.1152/japplphysiol.01046.2012 23305978

[43] Combs TP Berg AH Obici S Scherer PE Rossetti L. Endogenous glucose production is inhibited by the adipose-derived protein Acrp30. J Clin Invest (2001) 108:1875–81. 10.1172/jci14120 11748271 PMC209474

[44] Abdul-Ghani MA Jenkinson CP Richardson DK Tripathy D DeFronzo RA. Insulin secretion and action in subjects with impaired fasting glucose and impaired glucose tolerance: results from the Veterans Administration Genetic Epidemiology Study. Diabetes (2006) 55:1430–5. 10.2337/db05-1200 16644701

[45] Bock G Chittilapilly E Basu R Toffolo G Cobelli C Chandramouli V Contribution of hepatic and extrahepatic insulin resistance to the pathogenesis of impaired fasting glucose: role of increased rates of gluconeogenesis. Diabetes (2007) 56:1703–11. 10.2337/db06-1776 17384334

[46] Bock G Dalla Man C Campioni M Chittilapilly E Basu R Toffolo G Pathogenesis of pre-diabetes: mechanisms of fasting and postprandial hyperglycemia in people with impaired fasting glucose and/or impaired glucose tolerance. Diabetes (2006) 55:3536–49. 10.2337/db06-0319 17130502

[47] Viollet B Guigas B Sanz Garcia N Leclerc J Foretz M Andreelli F. Cellular and molecular mechanisms of metformin: an overview. Clin Sci (2012) 122:253–70. 10.1042/CS20110386 22117616 PMC3398862

[48] Knowler WC Barrett-Connor E Fowler SE Hamman RF Lachin JM Walker EA Reduction in the incidence of type 2 diabetes with lifestyle intervention or metformin. N Engl J Med (2002) 346:393–403. 10.1056/NEJMoa012512 11832527 PMC1370926

[49] Virtanen KA Lonnroth P Parkkola R Peltoniemi P Asola M Viljanen T Glucose uptake and perfusion in subcutaneous and visceral adipose tissue during insulin stimulation in nonobese and obese humans. J Clin Endocrinol Metab (2002) 87:3902–10. 10.1210/jcem.87.8.8761 12161530

[50] Marin P Rebuffe-Scrive M Smith U Bjorntorp P. Glucose uptake in human adipose tissue. Metabolism (1987) 36:1154–60. 10.1016/0026-0495(87)90242-3 3316924

